# Cytokines in Autoimmune Disease

**DOI:** 10.1155/2017/5089815

**Published:** 2017-07-11

**Authors:** Qingdong Guan, Xiaoling Gao, Junhui Wang, Yu Sun, Sudhanshu Shekhar

**Affiliations:** ^1^Cellular Therapy Laboratory, CancerCare Manitoba, Winnipeg, MB, Canada; ^2^Institute of Pharmacology, Taishan Medical University, Taian, China; ^3^The Institute of Clinical Research and Translational Medicine, Gansu Provincial Hospital, Lanzhou, China; ^4^Department of Physiology, University of Toronto, Toronto, ON, Canada; ^5^Department of Endocrinology & Metabolism, Qilu Hospital of Shandong University, Jinan, China; ^6^Department of Oral Biology, Faculty of Dentistry, University of Oslo, Oslo, Norway

The incidence and prevalence of autoimmune disease marked increased over the second half of the 20th century, and it has become a major health problem. These diseases are usually chronic and can be life-threating. The causes of autoimmune disease remain largely unknown. The recent advance in knowledge has greatly increased our understanding of pathogenesis of autoimmune disease, and it is widely accepted that environment, gene, and immunity contributed to the development of autoimmunity. Cytokines, including proinflammatory cytokines and anti-inflammatory cytokines, are important players in the pathogenesis of these diseases through multiple ways, such as regulating inflammation and angiogenesis.

In this special issue, we present original research articles as well as review papers on the role of cytokines in autoimmune diseases.

Psoriasis is a chronic and recurrent dermatitis, mediated by keratinocytes, T cells, and cytokines. In this special issue, P. S. S. de Oliveira et al. showed that the transcript levels of IL-17A, IFNG, and Foxp3, not IL-36A, IL-8, IL-33, IL-10, and IL-22, were increased in the moderate-severe patients with psoriasis. Some studies showed that psoriasis and rheumatoid arthritis (RA) had a common pathogenesis, but the precise molecular mechanism remains unclear. Through RNA sequencing of PBMC of psoriasis and RA patients and healthy controls, Y. Tan et al. showed that the common molecular mechanism of psoriasis and RA was characterized by a cytokine imbalance, in which ERK1/2, MAPK, TNF, CSF3, IL-6, IFN, and canonical signaling pathways played key roles in the pathogenesis of both diseases. The G*α*q-containing G protein (G*α*q) has been found to play an important role in immune regulation and development of autoimmune disease. D. Wang et al. showed that the expression of G*α*q was negatively correlated with the expression of IFN-*γ* in RA patients and further showed that G*α*q deficiency led to enhanced Th1 cell differentiation in animal study. G*α*q negatively regulated Th1 differentiation by modulating the expression of T-bet and activity of STAT-4. These results suggested the role of G*α*q in the pathogenesis of RA through modulating Th1 cells. The study by B. Wang et al. showed that serum IL-34 level was highly increased in RA patients; the interaction of IL-34 with IL-34R expressed on fibroblast-like synoviocytes (FLS) promoted IL-6 secretion by FLS, which further promoted the differentiation of Th17. Therefore, IL-34 might be involved in the pathogenesis of RA.

Cytokines drive and regulate multiple aspects of inflammatory bowel disease (IBD). Q. Guan et al. reviewed the recent advances of novel proinflammatory and anti-inflammatory cytokines found in IBD with focusing on IL-12 family (IL-12, IL-23, IL-27, and IL-35) and IL-1 family members (IL-1, IL-33, IL-36, and IL-37) as well as their relevance to the potential therapy of IBD. E. Vasilyeva et al. evaluated the difference of cytokine expression pattern in children and adult with Crohn's disease and found that at acute disease stage, all patients elevated serum levels of CXCL10; besides that, children patients increased serum levels of TNF and IL-6, while adult patient had elevated serum levels of GM-CSF and IFN-*γ*. B. C. de Sousa et al. explored the effects of *Morinda citrifolia* fruit juice in the treatment of DSS-induced murine colitis and showed that the treatment with this fruit juice played an important role in inhibiting intestinal inflammation during the development of murine colitis.

J. Ma et al. showed that the plasma IL-33 level was significantly increased in the patients of osteonecrosis of femoral head (ONFH) when compared to healthy controls, and interestingly, the level of IL-33 in the patients with late stage was higher than that in the patients with early stage, which indicated that IL-33 may be detrimental during ONFH.

In a review paper, C. Xiaoheng et al. presented a comprehensive summary of the association of various cytokines and related gene polymorphisms with susceptibility to autoimmune thyroid disease (AITD). The review focused on the structure and function of these cytokines and related genes in AITD and attempted to describe their differences in pathogenesis and clinical manifestations.

Adhesion molecules may play an important role in systemic lupus erythematosus (SLE) pathogenesis. But whether the expression of these adhesion molecules was affected by cytokines remains unclear. In this special issue, S. Lin et al. demonstrated that IL-15 significantly increased the expression of CD56 and CD11B but decreased the level of CD62L expression on NKT cells and NKT-like cells from SLE patients.

Different extra-articular clinical symptoms could appear in the course of spondyloarthropathy (SpA) disease, such as acute anterior uveitis (AAU), IBD, psoriasis, and psoriatic onychopathy. H. Przepiera-Będzak et al. showed that increased serum IL-18 and decreased serum endothelial-1 were associated with an increased risk of extra-articular symptoms in SpA patients; increased serum IL-6 and IL-23 also increased the risk of AAU. These results indicated that development of different extra-articular symptoms was relevant to elevated levels of different markers of inflammatory process.

One of the keys to control autoimmune disease is an improved understanding of cytokine responses in these diseases, which will aid the development of effective immunotherapeutic strategies. This special issue encompasses molecular mechanisms of cytokines in immunopathogenesis of autoimmune diseases from bench to bedside.

Overall, we believe that these articles may contribute to improve our knowledge of cytokine-mediated immune mechanisms in autoimmunity, to provide insights into designing of effective immunodiagnostic tools, and to present rational basis for the development of potential therapeutic agents.

